# Neuronal developmental gene and miRNA signatures induced by histone deacetylase inhibitors in human embryonic stem cells

**DOI:** 10.1038/cddis.2015.121

**Published:** 2015-05-07

**Authors:** K Meganathan, S Jagtap, S P Srinivasan, V Wagh, J Hescheler, J Hengstler, M Leist, A Sachinidis

**Affiliations:** 1 Center of Physiology and Pathophysiology, Institute of Neurophysiology and Center for Molecular Medicine Cologne (CMMC), Robert-Koch-Str. 39, Cologne, Germany; 2 Leibniz Research Centre for Working Environment and Human Factors, Technical University of Dortmund (IfADo), Dortmund, Germany; 3 Doerenkamp-Zbinden Chair for In Vitro Toxicology and Biomedicine, University of Konstanz, Konstanz, Germany

## Abstract

Human embryonic stem cells (hESCs) may be applied to develop human-relevant sensitive *in vitro* test systems for monitoring developmental toxicants. The aim of this study was to identify potential developmental toxicity mechanisms of the histone deacetylase inhibitors (HDAC) valproic acid (VPA), suberoylanilide hydroxamic acid (SAHA) and trichostatin A (TSA) relevant to the *in vivo* condition using a hESC model in combination with specific differentiation protocols and genome-wide gene expression and microRNA profiling. Analysis of the gene expression data showed that VPA repressed neural tube and dorsal forebrain (*OTX2*, *ISL1*, *EMX2* and *SOX10*)-related transcripts. In addition, VPA upregulates axonogenesis and ventral forebrain-associated genes, such as *SLIT1*, *SEMA3A*, *DLX2/4* and *GAD2*. HDACi-induced expression of miR-378 and knockdown of miR-378 increases the expression of *OTX2* and *EMX2*, which supports our hypothesis that HDACi targets forebrain markers through miR-378. In conclusion, multilineage differentiation *in vitro* test system is very sensitive for monitoring molecular activities relevant to *in vivo* neuronal developmental toxicity. Moreover, miR-378 seems to repress the expression of the OTX2 and EMX2 and therefore could be a regulator of the development of neural tube and dorsal forebrain neurons.

Detection of potential malformations of an organism upon developmental toxicant exposure during the development of the brain and peripheral nerve system may result in dyslexia, mental retardation, autism and cerebral palsy.^1^ However, detection of malformations in brain and peripheral nervous system requires animal models that have several disadvantages.^2, 3, 4, 5^ Although animals have traditionally been used for toxicity testing; a large number of animal requirement, false-positive results and above all interspecies differences require the implementation of new, human relevant, less time consuming and more cost-effective *in vitro* testing strategies. Moreover, animal studies are not ethically accepted by modern societies that urgently request reliable alternative *in vitro* systems.^2, 3, 4, 5^ The development of a reliable testing strategy to meet the increasing needs for toxicity testing is one of the main challenging issues of risk assessment.^6^

Embryonic stem cells (ESCs) are pluripotent cells that are capable of differentiating into any somatic cell types by a process orchestrated by highly hierarchical gene expression waves.^3, 7, 8^ The *in vitro* process of differentiation in human ESCs (hESCs) requires differentiation conditions that support the survival and maturation of the cells. hESCs have been used to generate different types of cells including neurons.^9, 10^ Therefore, hESCs based models are optimal for human relevant drug discovery and toxicity testing.^10, 11, 12, 13^ ESC-based differentiation systems toward neuronal, cardiac, hepatic and, in general, multiple lineage differentiation have been utilized to monitor the toxic nature of known developmental toxicants either on a mechanistic or functional level.^2, 3, 10, 14, 15, 16, 17^

Valproic acid (VPA) was effectively applied for the management of epilepsy and has been approved as an anticonvulsant drug since 1967. However, for more than a decade, many reports have provided evidence of fetal malformations induced by VPA application (reviewed in Cotariu and Zaidman^18^). VPA is a histone deacetylase inhibitor targeting histone deacetylase (HDAC), the enzyme that catalyzes the deacetylation of alpha-acetyl lysine within the core histone protein, and has also been therapeutically used for the treatment of cancer and other diseases. Other HDACi, such as trichostatin A (TSA) and suberoylanilide hydroxamic acid (SAHA), which are structurally similar hydroxamates, have been shown to cause the functional recovery of muscle dystrophy in mice and exhibit antirheumatic activity in mouse and rat models. The proposed mechanism of action of HDACi is the accumulation of acetylated histones and associated proteins involved in cell proliferation, cell migration, cell death and gene expression.^19, 20^ It has been reported that exposure to VPA during pregnancy causes many developmental abnormalities, including neural tube defects (mostly spina bifida), cardiac, skeletal, and limb defects, and fetal valproate syndrome.^18, 21, 22^ The interspecies analysis of VPA exposure during pregnancy showed a posterior neural tube closure (known as a spina bifida aperta) and an anterior neural tube closure (known as exencephaly) in humans and mice, respectively.^23^ VPA targets genes regulating neural tube development and closure and deregulates gene expression by inhibiting HDAC, which may represent an important key to the teratogenesis.^24^ Several *in vivo* studies have demonstrated that HDACi cause developmental defects during embryonic development, particularly delayed gastrulation, the perturbation of mesoderm formation, and reduced mid-trunk and posterior formation.^25^

There is increasing evidence that microRNAs (miRNAs) and non-coding RNAs regulate the post-transcriptional processing of mRNAs and have a definitive role in development, as first demonstrated by the lin-4 miRNA, which was shown to have a role during larval stage development in *Caenorhabditis elegans* and to have a major role in the transcriptional regulatory network during development.^26, 27^ Many miRNAs, including miR-124a, miR-125, miR-132 and miR-219, are emerging as essential regulators during the cell lineage commitment toward neuronal development.^28, 29^ Each miRNA can target hundreds of mRNAs, frequently in combination with other miRNAs.^30^ Therefore, the application of gene expression analyses in conjunction with miRNA expression may have a significant impact for the discovery of new predictive developmental toxicity pathways and biomarkers. The deregulation of miRNA and gene expression networks in response to a toxicant is often epigenetically regulated and it is controlled by histone modification or methylation.^31, 32^ In this study, we combined transcriptomics with respect to mRNA and miRNA expression with a multilineage differentiation hESC model that mimics developmental processes under *in vivo* conditions to predict adverse ectodermal developmental effects of HDACi observed under *in vivo* conditions. Importantly, this study unveils that HDACi, especially VPA dysregulating forebrain specification through miR-378.

## Results

### Determination of sublethal concentrations and HDAC activity assay

**Figure 1:**
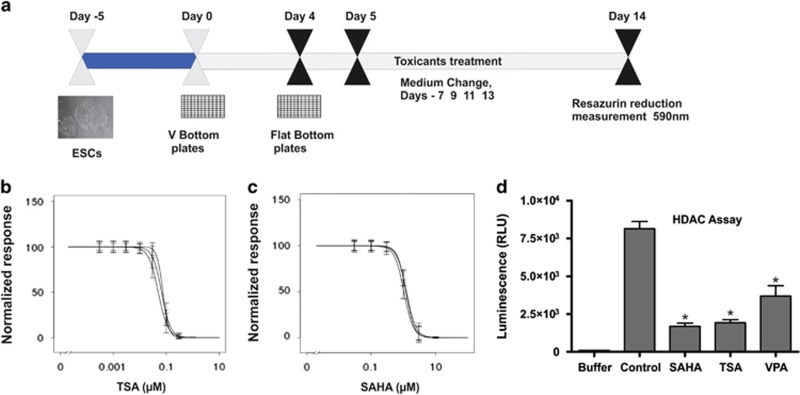
Determination of sublethal concentration and HDAC inhibition. (**a**) To determine the dose-response curve for TSA and SAHA, hESCs were exposed to various concentrations of the compounds from days 5 to 14. Resazurin reduction was used as a parameter to detect cytotoxicity. Cell viability was calculated after normalizing the fluorescence intensity values to the control. Three independent biological replicates were performed, and at least five technical replicates were performed for each biological replicate. The inhibitory concentration (IC) values were calculated from the graph. (**a**) A representative scheme for sublethal concentration determination. (**b** and **c**) Cytotoxicity curve of TSA and SAHA based on the resazurin reduction assay. (**d**) Enzymatic activity of VPA, SAHA and TSA compared with control. Data represent mean values of three measurements±S.E.M (*P<0.05, SAHA, TSA and VPA *versus* control). RLU, relative luminescence units

Sublethal concentrations for TSA and SAHA were determined according to the exposure protocol shown in Figure 1a. For VPA, we used sublethal concentrations of 2 mM, as previously applied.^2^ The calculated sublethal dose of VPA (2 mM), TSA (0.02 *μ*M; Figure 1b) and SAHA (0.5 *μ*M; Figure 1c) were used for global gene expression profiling. To test whether VPA, SAHA and TSA inhibited HDAC (HDAC class I and II enzymes), 14 days untreated and HDACi-treated embryoid bodies (EBs) were assayed for HDAC activity (Figure 1d). As compared with the HDAC activity of the untreated 14-day EBs, treatment with TSA, SAHA and VPA significantly reduced the HDAC activity.

### Genome-wide expression profiling of HDAC inhibitors

**Figure 2:**
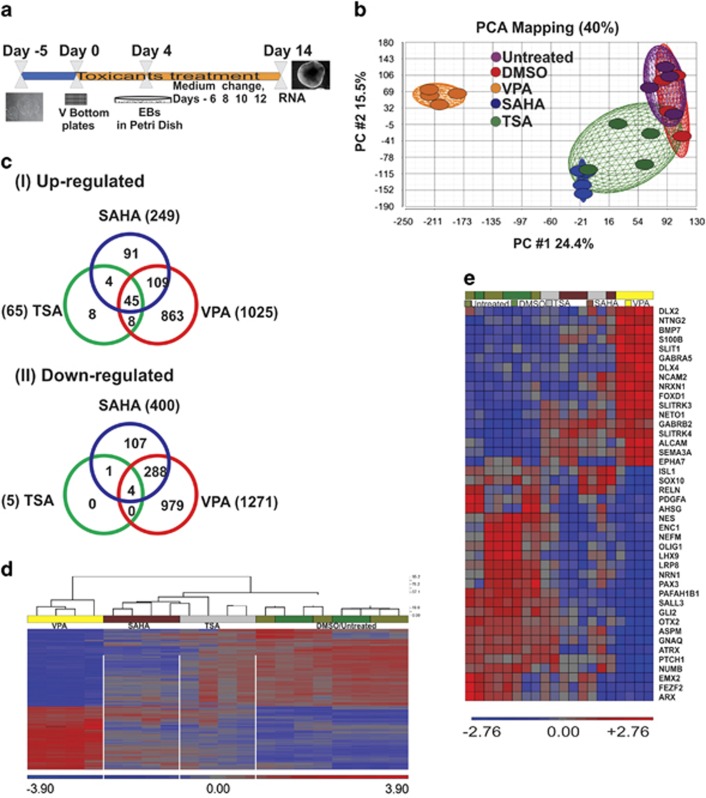
Gene expression changes in hESCs differentiation after exposure to the toxicants for 14 days. (**a**) Schematic representation of hESCs differentiation and compound treatment for gene expression. Treatment was performed from days 0 to 14 during differentiation, and samples were processed for microarray experiments. (**b**) The PCA plot shows the variations in the gene expression levels and the induced changes in the expression patterns. The four biological replicates for each sample group are shown in single colors. (**c**) Venn diagram showing the number of differentially regulated transcripts for upregulated (I) and downregulated (II) transcripts for each compound. (**d**) The hierarchical clustering of DEGs shows a differential expression pattern of VPA in comparison with that of SAHA and TSA, which shows a similar gene expression patterns compared with the controls. The data represent from four biological replicates. (**e**) An expression heat map shows a similar gene expression pattern by HDACi VPA, SAHA and TSA compared with the DMSO-treated and/or untreated control cells. The up- and downregulated genes are represented by red and blue colors, respectively. The scale bar represents the fold change for each compound relative to the controls

To identify differentially regulated genes, we differentiate hESCs through multilineage differentiation for 14 days, as described in the Materials and methods section. The compound exposure and sample collection for gene expression profiling is shown in Figure 2a. Principal component analysis (PCA) showed a grouping between the untreated and 0.01% dimethyl sulfoxide (DMSO)-treated cells. In contrast, only SAHA and TSA, both being structurally similar hydroxamates, grouped together among the HDACi. The number of differentially expressed genes (DEGs) after TSA and SAHA applications was relatively low compared with the high number by the VPA treatment and the untreated/DMSO-treated groups. The higher the distance of the treated cells from the control cells, the more DEGs were found between the cell populations. Altogether, PCA showed 40% variability of the gene expression level. PC1 at 24.4% mainly accounted for the VPA treatment (Figure 2b). Among the treatments, VPA resulted in a larger number of DEGs. In comparison with the untreated cells, 649, 2296 and 70 genes were found to be significantly regulated by SAHA, VPA and TSA, respectively. To reveal a common regulation pattern among the HDACi, we generated a Venn diagram for the up- and downregulated DEGs, showing 45 and 4 common regulated transcripts, respectively (Figure 2c). The complete list of DEGs is shown in Supplementary Table S2 (A–C). Among the HDACi, VPA and SAHA shared a larger number of common DEGs compared with TSA. Overall, the number of downregulated genes was higher compared with upregulated DEGs (Figure 2c). We used a hierarchical clustering analysis to visualize similar expression patterns of well-known positive compounds, such as VPA, compared with the other compounds. The 2296 DEGs for VPA treatment showed a close similarity to SAHA and were significantly different when compared with the untreated and DMSO-treated cells (Figure 2d). To further investigate the relationship among HDACi, the dysregulated genes, and the associated GO categories, we examined the similarly regulated genes between the three compounds (Figure 2e) and identified genes related to neuronal development. The heat map in Figure 2e shows that VPA induced the expression of ventral forebrain markers such as *DLX2*, *DLX4* and GABAergic genes including *GABRA5* and *GABRB2*. In contrast, the expression of dorsal forebrain-related genes such as *PAX3*, *OTX2* and *ISL1* was repressed. Especially the genes such as *GABRAB2*, *NES* and *OLIG1* were repressed by all the three compounds.

### Analysis of DEGs altered in response to VPA

**Figure 3:**
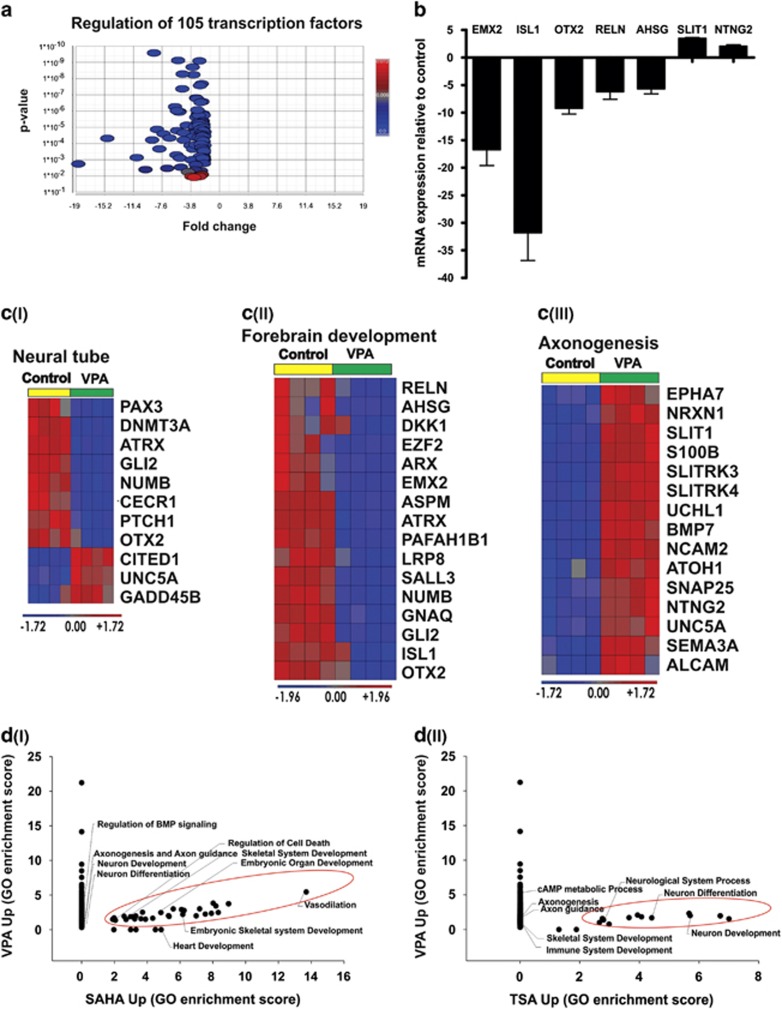
VPA treatment targets major transcription factors that are essential for neural development, including neural tube-related, forebrain development-related and axonogenesis-related genes. (**a**) Volcano plot showing the significant expression pattern of 105 transcripts derived from downregulated DEGs by VPA treatment. The *x* axis shows the fold change and the *y* axis shows the *P*-values. The scale bar indicates the *P*-values. (**b**) The representative genes for forebrain development and axonogenesis were analyzed using RT-qPCR. The mRNA expression values are relative to the untreated control. The error bar shows the S.D. from three technical replicates. (**c**) Signal intensity plots showing the gene expression pattern of selected genes for (I) neural tube, (II) forebrain development and (III) axonogenesis. (**d**) The enrichment score values of GO BPs altered by VPA *versus* SAHA (I, upregulated), VPA *versus* TSA (II, upregulated) (*P*<0.05) are illustrated in a scatter plot. The enrichment score values of the respective treatment are indicated on the *x* and *y* axes. The common BPs are highlighted in ovals

*In vivo, in vitro* and clinical studies have proven that VPA induces severe neurological developmental defects.^13, 33, 34, 35^ Taking this in consideration, we used VPA to demonstrate whether the adverse effects observed *in vivo* may be also recapitulated to some extent the *in vitro* conditions using the hESC differentiation model. To identify the functional relevance of DEGs because of VPA, we separately analyzed the up- and downregulated DEGs using the database for annotation, visualization and integrated discovery (DAVID) gene ontology (GO) analysis, as mentioned in the Materials and methods section. The downregulated gene biological processes (BPs) encompassed 105 transcription factors, including many neuronal development-related transcription factors, such as *HES5, ISL1, OTX2, LHX9, REST, DNMT3A* and *ATRX*. BPs related to neuronal development, including forebrain development, cerebral cortex development and neuronal differentiation, are shown in Table 1a. In contrast, the upregulated BPs were predominantly axonogenesis and vasculature development, as shown in Table 1b. The significant expression pattern of the 105 transcription factors is shown as a volcano plot in Figure 3a. Representative genes (up- and downregulated) identified by the microarrays as differentially expressed were validated by real-time quantification PCR (RT-qPCR; Figure 3b). One of the prominent effects of VPA was the perturbation of the expression of neural tube-associated genes that has been consistently reported in clinical and *in vivo* studies.^23, 35, 36^ Accordingly, neural tube-relevant genes, such as *DNMT3A, ATRX, GLI2* and *OTX2*, were found to be downregulated (Figure 3c (I)). Sixteen genes, including the *ISL1, OTX2, SALL3, EMX2* and *ATRX* transcription factors involved into forebrain development were downregulated (Figure 3c (II)). Accordingly, a perturbation of the expression of these genes under *in vivo* conditions has been reported.^37^ VPA treatment resulted in the upregulation of axon guidance molecules including, *SLIT1, NTNG2, SEMA3A* and *BMP7*, which are related to the axonogenesis BP (Table 1b) and the expression pattern of the genes were represented as a heat map in Figure 3c (III). A GO analysis for the three compounds identified 31 and 35 enriched BPs down- and upregulated, respectively, by both VPA and SAHA and 14 enriched BPs that were upregulated by both VPA and TSA. The complete GO results are shown in Supplementary Tables S4A and E. VPA as a standard reference compound showed unique and common BPs for up- and downregulated transcripts based on the GO enrichment score. Common and different BPs among VPA, SAHA and TSA are represented as a scatter plot. As shown, GO BPs enriched for VPA- and SAHA-upregulated genes included vasodilation and embryonic organ development (Figure 3d (I)). Further common GO BPs enriched for genes for upregulated by VPA and TSA included neurological system process and neuronal differentiation (Figure 3d (II)).

**Figure 4:**
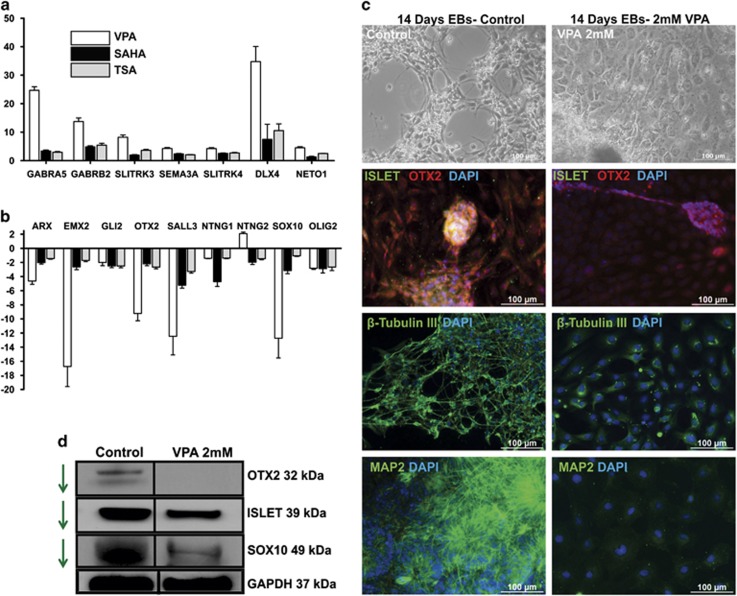
VPA treatment represses the dorsal forebrain transcription factors and enhances the ventral GABAergic neuronal markers. The representative (**a**) up- and (**b**) downregulated genes were analyzed using RT-qPCR. The mRNA expression values are relative to the untreated control. The error bars show the S.D. from three technical replicates from an independent experiment. (**c**) Representative morphology of cells after VPA treatment as compared with untreated cells, which shows neuronal cells. Detection of the neuronal-specific transcriptional factors (ISLET and OTX2) and neuronal-specific cytoskeleton proteins (*β*-tubulin and MAP2) by immunocytochemistry. The protein concentration is reduced in VPA-treated cells. (**d**) Immunoblotting analysis of neuronal-specific transcriptional factors. The arrow shows a reduced level in of VPA-treated cells

Selected neuronal development genes up- (*GABRA5, SLITRK4, DLX4* and *NETO1*) and downregulated (*ARX, EMX2, SALL3* and *SOX10*) were validated by RT-qPCR for all three compounds (Figures 4a and b). Morphological analysis showed that 14 days differentiation exhibits many neuronal projections, whereas for VPA treatment, the cells are flat and the neuronal projections are absent (Figure 4c). To find out the cellular localization of the forebrain development-related genes and mature neuronal-specific markers such as OTX2, ISL1, *β*-tubulin III and MAP2, immunocytochemistry (Figure 4c) was performed. To determine the effect of VPA at the protein level, the selected neuronal-relevant proteins were analyzed by western blotting (Figure 4d). The interactions between the genes were identified using MetaCore database (description mentioned in Materials and methods section). From the negatively regulated VPA signatures, we found development of general neurogenesis process network (Supplementary Figure S1). The network shows the essential neuronal-relevant transcription factors and its interaction partners.

### miRNA profile during VPA treatment of hESCs during multilineage differentiation

**Figure 5:**
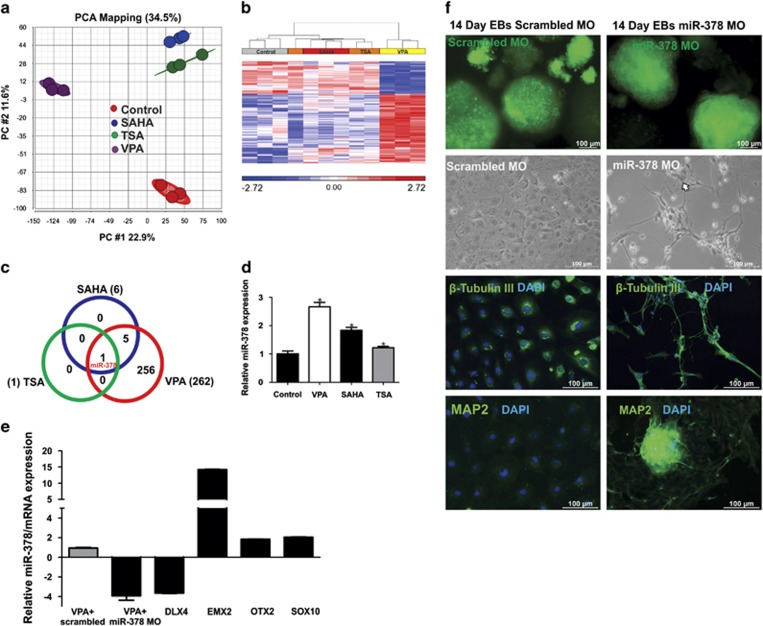
HDAC inhibitors commonly dysregulated miR-378 and VPA represses dorsal forebrain markers via mir-378. (**a**) PCA of normalized miRNAs demonstrates total 34.5% variance. (**b**) Hierarchical cluster analysis of differentially expressed miRNAs (FDR *P*<0.05, ±1.5-fold change) after HDAC inhibitors treatment. Red color showed upregulated and blue color showed downregulated miRNAs. VPA shared a higher percentage of variances compared with TSA and SAHA. (**c**) The Venn analysis showed that miR-378 is commonly dysregulated within the HDACi. (**d**) RT-qPCR analysis of miR-378 for HDACi is consistent with the microarray results. The validation has been performed with the same array samples from three biological replicates. The error bar represents S.D. (**e**) In all, 75% of miR-378 knockdown enhances the dorsal forebrain markers and represses the ventral forebrain markers. Error bar represents S.E.M. from two biological replicates (values were set as relative fold changes of the mRNA levels as compared with the VPA control). (**f**) Fluorescence microscopy (excitation: 488 nm, emission: 509 nm) demonstrating cellular uptake of the scrambled and the MOmiR-378. Morphological representation at day 14 shows miR-378 knockdown exhibits appearance of neuronal projections and expression of mature neuronal-specific markers even after 2 mM VPA treatment

During the course of multilineage differentiation, HDACi were treated up to 14 days to identify differentially expressed miRNAs. The tissue-specific expression of miRNAs during differentiation and developmental processes may have a significant role in the regulation of neuronal development and neurtoxicity.^27, 38^ We utilized the same source of RNA for the miRNA assessments, as mentioned in the Materials and methods section. The differentially regulated analysis for VPA (compared with the control) resulted in 256, 6 and 1 human miRNAs at ±1.5-fold change (Supplementary Table S3) for VPA, SAHA and TSA, respectively. The PCA analysis showed TSA in proximity to the control, which showed 22.9% variance for PC1 and 11.6% variance for PC2 (Figure 5a). The differentially regulated miRNAs were represented as hierarchical clustering (Figure 5b). Among three compounds, VPA exhibits more number of differentially regulated miRNAs compared with SAHA and TSA (Figure 5b). To find out common and different miRNAs among three compounds, we performed Venn analysis and strikingly miR-378 was commonly regulated by all of the three HDAC inhibitors (Figure 5c). To validate the miR-378 expression, we performed RT-qPCR analysis and the expression pattern is consistent with the array results (Figure 5d). To find out whether expression of miR-378 is relevant to the repression of dorsal forebrain mRNAs, we performed the knockdown of miR-378 for VPA using an antisense morpholino oligonucleotide (MO) targeting the miR-378 (miR-378 MO). The RT-qPCR analysis showed miR-378 knockdown overexpresses the dorsal telencephalon genes such as *EMX2*, *OTX2* and *SOX10*, whereas the ventral telencephalon gene *DLX4* was repressed (Figure 5d). Also shown in Figure 5f is the cellular uptake of miR-378 MO and scrambled MO. The morphological analysis showed VPA treatment inhibits the neuronal projections, whereas after miR-378 knockdown neuronal extensions were observed (Figure 5f). In general, knockdown of the miR-378 counteracted the effects of VPA on the gene expression level of *OTX2* and *SOX10* (Figure 5e).

## Discussion

Recently, we established hESC multilineage differentiation test system that allows the detection of perturbations in differentiation processes toward to the three germ layers, endoderm, mesoderm and ectoderm.^2, 3, 10^ Applying our test system, we were able to demonstrate that cytarabine at a low concentration (1 nM) induced the ectoderm lineage and in parallel inhibited the mesodermal lineage.^10^ Interestingly, using thalidomide, our test system monitored perturbations in the mesodermal lineage, such as limb and heart development, as observed in humans.^3^ Moreover, we also established hESC-based differentiation test system allowing the detection of early neurogenesis.^2, 39^

In this study, three HDACi compounds were tested using our multilineage differentiation test system and the resulting gene signatures were compared. Sublethal doses of the selected compounds were tested during differentiation for global gene expression profiling. This work shows that TSA, SAHA and VPA are effective inhibitors of HDACs class I and II enzymes at an IC_10_ value used for microarray experiments. A subsequent statistical analysis of DEG expression showed a higher number of DEGs regulated for the well-known positive compound VPA compared with the other compounds. HDAC inhibition has been proven as a molecular target and cause of teratogenicity because of VPA, classifying it as an HDACi.^40, 41^ Although we have observed almost equal HDAC inhibition for all the three HDACi, the gene expression profiling for VPA showed a more potent dysregulation in the stem cell differentiation compared with TSA and SAHA. HDACi have been studied in both self-renewal, induced pluripotent cells (iPSC) generation and in differentiation context of stem cells with similar observation.^42, 43, 44^ VPA shows 100-fold more efficient in iPSC generation as compared with TSA and SAHA.^45, 46^ This suggests that VPA could act via alternate signaling pathways other than HDAC I and II and can be the reason for potent dysregulation of stem cell differentiation as compared with TSA and SAHA. In addition, it was demonstrated that VPA augments neuronal differentiation but disrupt astrocyte and oligodendrocyte differentiation through HDAC inhibition.^47^

Neuronal differentiation is a very dynamic process that involves epigenetic mechanisms such as DNA methylation, which is governed by DNA methyl transferases (DNMTs), recruitment of the methyl-CpG binding domain (MBD), and histone modification.^48, 49^ VPA repressed such epigenetic regulators as *ATRX*, *SUZ12*, *MLL*, *TRDMT1*, *MBD2* and *DNMT3A* potentially the reason for the disruption of neuronal development, resulting in VPA-mediated teratogenicity (Supplementary Table S2A). Several mouse and human studies have proven that consumption of VPA during pregnancy causes spina bifida and exencephaly, both representing neural tube defects.^23, 35, 36^ Many signaling pathways such as the sonic hedgehog, WNT pathways and epigenetic transcriptional regulators, such as *PAX3, CITED1*, *SUZ12*, *ATRX*, *PTCH1* and *GLI2* are essential for neural tube closure (reviewed in Copp and Greene^50^).

The downregulation of these critical transcriptional factors by VPA observed in this study can partially recapitulate the neural tube defect effects of VPA during early development (Figure 3c (I)). In addition to neural tube defects, mouse embryos exposed to VPA show a significant disorientation of the neuroepithelium; in particular, defects were observed in the forebrain region, including the flattened appearance of telenchephalic hemispheres.^33, 37^ The expression of *OTX2* in the visceral endoderm normally maintains the development of the anterior neuroectoderm, and the knockout of *OTX2* keeps the endoderm active cells distal when a primitive streak is formed, thus suggesting that *OTX2*-positive distal visceral endoderm cells are required for the generation of the forebrain. Indeed, a homozygous mutation analysis showed that a lack of *OTX2* resulted in the absence of forebrain, midbrain and dorsal head development, thus confirmed that it is essential for dorsal forebrain development.^51, 52, 53^ The expression of *EMX2* is essential for dorsal telencephalon development, whereas mutant forebrain showed reduced cerebral hemispheres, and the roof between the cerebral hemispheres was expanded.^54^ This study also revealed disruption of SOX10 expression by VPA, which is expressed during the neural crest and glial lineage development. Neural crest cells emerged from the dorsal region of the neural tube thereby further proceed into epithelial-to-mesenchymal transition then migrate into different parts of the body especially neurons and glia. There is enough evidence suggesting that epigenetic and transcriptional activities are involved in the neural crest development and the SOX10 expression in the dorsal neural tube is acting as a top of the gene regulatory network during the neural crest epithelial-to-mesenchymal transition, migration and differentiation.^55, 56, 57, 58, 59^ In another study, TSA induced neural tube defect through dysregulation of neural crest markers including SOX10.^60^ This study provides additional evidence that VPA induces neural tube defect through repression of SOX10.

The homeobox genes *DLX2* and *ARX* are expressed in subpallium, and homozygous mutants of *DLX2* die at birth because of subtle forebrain defects. *DLX2* and *ARX* act as transcriptional targets for Neurophilin-2 and *PAX4,* respectively, during forebrain development (reviewed in Wigle and Eisenstat^61^). In the forebrain, there are two primary compartments, the thalamus and pretectum, and the expression of *LHX9* is needed for defined neuronal thalamus differentiation.^62^ The GO analysis of downregulated DEGs because of VPA in our study showed an enrichment of forebrain development BPs (Table 1a), with 16 transcripts for transcription factors *OTX2*, *ARX*, *LHX9*, *ISL1*, *GLI2*, *SALL3*, *ATRX* and *EMX2* (Figure 3c (II)). A similar expression pattern was observed for the other HDACi, TSA and SAHA (Figure 5a). VPA has been used as a mood stabilizer by increasing GABAergic activity at synapses, and studies have demonstrated that VPA induces a disturbance in excitatory and inhibitory neuronal activities by interfering with GABA receptors in the hippocampus and cortical neurons.^63, 64, 65^ GABAergic interneurons expresses markers such as DLX1/2, GAD1/2 and GABRA5 during the ventral forebrain specification and maturation.^66^ The HDACi tested in this study upregulated *GABRA5* and *GABRB2*, which may explain the activation of GABAergic neuronal genes during hESCs differentiation. In an *in vivo* study, the administration of HDACi significantly recovered stroke injury through the amplification of myelinated axonal density and neurogenesis.^67^

Axonogenesis-related genes (Table 1b) and axon guidance genes, such as *SLITRK3*, *SLITRK4* and *SEMA3A*, were identified for the first time to be overexpressed by VPA treatment (Figure 3c (III). Notably, our transcriptome data clearly suggest that, among the three germ layers, HDACi inhibitors cause perturbations mainly in the differentiation of the ectoderm to neuronal lineage. Moreover, among neuronal cell lineages, neural tube and dorsal and ventral forebrain-related transcripts were specifically disrupted. Further in depth studies could reveal whether the augmentation of ventral neurons is at the expense of the dorsal forebrain markers.

miRNAs have an emerging role in the regulation of gene expression and regulate such BPs as development, immune responses and metabolism; it has been recently recognized that miRNAs are also key regulators of drug toxicity.^68, 69^ Accordingly, it is imperative to identify miRNA signatures for HDACi and its relevant mRNA targets to uncover its developmental toxicity. With regard to VPA treatment, the principal downregulated miRNA cluster encompassed miR-302a, b, c and d, which are well proven as markers for self-renewal and/or proliferation; consequently, VPA downregulated the pluripotency markers *POU5F1*, *NANOG* and *LIN28* >7-fold (Supplementary Table S2A).^70, 71, 72^ Most importantly, we found all the three HDACi overexpressed miR-378 in this study. Previously, miR-378 has been studied in the context of cell survival, colony formation and tumor growth through direct inhibition of vimentin (*VIM*).^73^ *VIM* expresses during the astroglial specification and it was proved that by modulating *VIM* and astroglial population, neurogenesis has been significantly increased.^66, 74^ VPA treatment increases miR-378 and represses *VIM* in this study and further to examine the effect of miR-378 expression, we performed transient knockdown of miR-378. Surprisingly, knockdown of miR-378 reverses the expression of *OTX2*, *EMX2*, *SOX10* and *DLX4* at mRNA level. These findings suggest an important role of miR-378 for the process of neurogenesis.

In summary, this study demonstrates that a multilineage *in vitro* hESC differentiation model in combination with transcriptome studies can, to some extent, recapitulate the developmental neurotoxicity of HDACi observed under *in vivo* conditions. In particular, the well-established neural tube and forebrain development defects because of VPA were recapitulated by monitoring the significant inhibition of several known transcription factors involved in the development of the neural tube and forebrain. Notably, the role of VPA in axonogenesis and repression of forebrain markers through miR-378 has never before been demonstrated by any *in vitro* study. Altogether, our multilineage differentiation testing system can be applied for both toxicity screening and to uncover new molecular developmental mechanisms.

## Materials and Methods

The compounds VPA, TSA and SAHA were purchased from Sigma, Steinheim, Germany. The VPA stock solution was prepared with water; for the remaining compounds 10 mM/20 mM/100 mM stock solutions were prepared using DMSO. DMSO was added to the solvent control medium at a final concentration of 0.005%.

### Sublethal dose determination (cell viability assay) and HDAC activity assay

The sublethal dose of the compounds was determined by using the CellTiter-Blue cell viability assay (Promega, Mannheim, Germany). hESCs was differentiated according to our recent description.^3^ On day 4, EBs were manually picked and seeded (two EBs per well) in flat-bottom 96-well black plates with a differentiation medium (see the culture conditions below for the components). On day 5, the drug-containing medium was added. The medium was replenished every other day, and at least eight concentrations were selected for each drug; at least five technical replicates were maintained for each concentration and the experiments were repeated as three independent biological replicates. On day 14, 20 *μ*l of the CellTiter-Blue (Promega) reagent was applied with 100 *μ*l of differentiation medium. After 90 min at 37 °C and 5% CO_2_, the fluorescence was measured at an excitation wavelength of 560 nm and an emission wavelength of 590 nm with a Teccan (Carlsheim, Germany) fluorescent reader. The concentrations used in this study were the highest non-cytotoxic concentrations (designated here as the benchmark concentration, BMC) experimentally determined by exposing cells to a large range of test concentrations and then recording their viability using a resazurin reduction assay.^2^ BMC was defined as the calculated concentration that led to a reduction in viability of 10%. For the calculation, the viability data were normalized to a solvent control, and the data were represented (logarithmic plots) as nonlinear sigmoidal curves using GraphPad Prism (La Jolla, CA, USA), version 4.01. The curves were averaged and BMC was determined as the *x*-value corresponding to the *y*-value of 90%.

The HDAC inhibition was determined using HDAC-Glo I/II assay (Promega) on day 14 control EBs. The attached EBs were exposed at BMC of respective compounds and incubate at 37 °C and 5% CO_2_, for 1 h. After 1 h of incubation, 100 *μ*l of the HDAC-Glo I/II Reagent (Promega) was added to each well and incubated at room temperature for 60 min. After 60 min, the luminescence was measured with a Tecan fluorescent reader. Inhibition activity of the compound was calculated by subtracting compound luminescence value from control.

### Cell culture conditions and cell differentiation for gene expression

Human ESCs were cultured and differentiated as we described previously.^3, 10^ Briefly, NIH-registered H9 hESCs (WiCell, Madison, WI, USA) were cultured in DMEM-F12, 20% KO serum replacement, 1% non-essential amino acids, penicillin (100 units/ml), streptomycin (100 mg/ml) and 0.1 mM *β*-mercaptoethanol, supplemented with 4 ng/ml basic fibroblast growth factor (bFGF). Before initiating differentiation, the cells were maintained for 5 days under feeder-free conditions using the mTESR1 medium. For multilineage differentiation, EBs were formed according to previous description.^3^ Briefly, on day 0, approximately 70–80 clumps were seeded on Pluronic-coated V-bottom plates and incubated in differentiation medium (H9 growth medium without bFGF) either in the presence of a test compound and/or DMSO/untreated. The coating was performed at least 45 min before the experiments, and the Pluronic was completely removed. On day 4, EBs were manually removed and collected in non-adherent plates and maintained in the respective compound/DMSO/untreated medium; the culture medium was replenished with fresh medium every other day. On day 14, the samples were collected for gene expression profiling.

### Microarray analysis

For the microarray analysis, samples, drug treatment, solvent and untreated controls from four independent biological replicates (*n*=4), were collected on day 14. The RNA isolation, microarray labeling and hybridization techniques were followed as previously reported.^3^ Briefly, total RNA, including small nucleotide RNA, was isolated using TRIzol reagent (Invitrogen, Darmstadt, Germany) and CHCl_3_ (Sigma) and further purified with the miRNeasy mini kit (Qiagen, Hilden, Germany). The quantification and quality control measurements were performed using a NanoDrop spectrophotometer (ND-1000, Thermo Fisher, Langenselbold, Germany) and an Experion automated electrophoresis system (Bio-Rad, Munich, Germany), respectively. For the microarray labeling, 100 ng total RNA was used as the starting material; after amplification, 12.5 *μ*g amplified RNA was hybridized to Affymetrix Human Genome U133 Plus 2.0 arrays (Affymetrix, Santa Clara, CA, USA). The Affymetrix HWS kit and Genechip Fluidics Station-450 were used according to the manufacturer's instructions for the washing and staining steps. After staining, the arrays were scanned using an Affymetrix Gene-Chip Scanner-3000-7G, and the Affymetrix GCOS software was used for the quality control analysis. The same total RNA from the untreated controls and VPA-treated cells (*n*=3) were used for the miRNA expression profiling. For labeling, 700 ng of total RNA was used as the starting material, and the Affymetrix FlashTag Biotin HSR RNA Labelling kit was used for miRNA ligation. The labeled samples were hybridized to the Affymetrix miRNA 3.0 arrays for 16 h at 48 °C in an Affymetrix hybridization oven 645. After incubation, the arrays were washed and stained in the fluidics station 450 using fluidics script FS450_0002 and scanned using an Affymetrix Gene-Chip Scanner-3000-7G. All the arrays passed the Affymetrix quality control analysis performed by the Affymetrix Expression console (version 1.2).

### Statistical data and functional annotation analysis of gene arrays

The microarray statistical data analysis and visualization were performed using the Partek Genomics suite version 6.6 (Partek, St. Louis, MO, USA). For mRNA arrays, the probes set intensity values were generated by RMA background correction, quantile normalization, log2 transformation and median polished probe set summarization. The normalized probe sets were used for the generation of a PCA, and a one-way ANOVA model was used to generate the differentially regulated genes with at least a twofold change using a Benjamini and Hochberg FDR correction at *P*≤0.05. For the miRNA arrays, the probe set intensity values were generated from RMA normalization, and a one-way ANOVA model was utilized for the determination of the differentially regulated genes at 1.5-fold change with a Benjamini and Hochberg FDR correction at *P*≤0.05. The differentially regulated genes were used as an input for DAVID bioinformatics tools to decipher the GOsgene ontologies. To build the gene network from the differentially regulated genes, we used MetaCore (Thomson routers) data mining and pathway analysis database. Briefly, Metacore is a manually curated high-quality species-specific database from transcription factors, ligands, kinases and their interactions are represented on pathways and networks.

### Real-time quantification PCR

The same source of RNA used for the microarray experiments was utilized for the RT-qPCR validation. For cDNA synthesis, 700 ng total RNA was used as the starting material with the Super Script Vilo cDNA synthesis kit (Invitrogen, Darmstadt, Germany) according to the kit instructions. cDNA was diluted with nuclease-free water, and 100 ng of cDNA was used as the starting material for RT-qPCR. The primer sequences were procured from Origene (www.Origene.com). Platinum SYBR Green qPCR SuperMix (Invitrogen, Darmstadt, Germany) was used for the PCR assays with the Applied Biosystems (Darmstadt, Germany) 7500 FAST cycler. The gene expression of target genes was normalized to the reference gene GAPDH. The mRNA expression values were represented as the fold change relative to the respective control. The primer sequences are listed in the Supplementary Table S1. TaqMan MicroRNA Assays (Applied Biosystems) were performed for miRNA 378 expression using hsa-miR-26b (TM-000407) and hsa-miR-378 (TM-000567). Reverse transcription was carried out using the TaqMan MicroRNA Reverse Transcription Kit (P/N 4366596) and the PCR was performed with 15 ng RNA as a template, TaqMan Universal PCR Master Mix, No AmpErase UNG (P/N 4324018, Applied Biosystems) and TaqMan Assay miRNA Mix (Applied Biosystems) as recommended by the manufacturer.

### Western blotting and immunostaining

Western blot analyses were performed with 10 *μ*g protein as described previously.^75^ Total protein extracts were separated by SDS-polyacrylamide gel electrophoresis and blotted onto polyvinylidene fluoride membranes (Invitrogen, Karlsruhe, Germany). After blocking the membranes with 5% non-fat milk suspended in T-PBS (0.1% Tween 20, Sigma-Aldrich, Seelze, Germany), the membranes were incubated with the following primary antibodies in 1% non-fat milk at 4 °C overnight: anti-OTX2 (Abcam, Cambridge, UK, ab130238), anti-ISLET 1 (Abcam, ab109517), anti-SOX10 (Abcam, ab155279) and anti-GAPDH (Abcam, ab9485). The proteins were visualized using the ECL Pierce Fast Western Blot system (Thermo Fisher Scientific, Schwerte, Germany, 35050).

### Immunocytochemistry

For immunocytochemistry analyses, day 12 EBs (control, VPA and hsa-mir-378-treated group) were plated on fibronectin-coated coverslips. On day 14, the EBs were fixed with 99% methanol (Roth, Karlsruhe, Germany) at −20 °C for 10 min. Thereafter, cells were permeabilized with 0.3% Triton X-100 (Sigma-Aldrich) at room temperature for 20 min. Cells were blocked with 5% bovine serum albumin (PAA, Pasching, Austria) and stained with anti-ISLET 1 (ab109517, Abcam 1 : 200), anti-OTX2 (ab130238, Abcam 1 : 200), anti-MAP2 (M9942, Sigma, Seelze, Germany, 1 : 200) and anti- *β*-tubulin III (T2200, Sigma, 1 : 400). Antibodies were used at dilution as recommended by the manufacturer. Primary antibodies were detected with species matched Alexa-488 (Invitrogen, Grand Island, NY, USA), Alexa-568-conjugated secondary antibodies and DAPI (Invitrogen, Grand Island, NY, USA). Images were taken using an Axiovert 200 microscope and Axiovision 4.3 software (Carl Zeiss, Oberkochen, Germany).

### MO of hsa-mir-378

MOs of has-mir-378 (Gene Tools, LLC, Philomath, OR, USA) complementary to specific sequence from 5′ to 3′ ACACACAGGACCTGGAGTCAGGAGC and non-target sequence (scrambled) CCTCTTACCTCAGTTACAATTTATA were used. The MO sequences were selected on the basis of the manufacturer's recommendations (25 nucleotides antisense). In all, 15 *μ*M of oligonucleotide was treated on the EBs from days 10 to 14 in presence of VPA. Day 14 EBs were processed for RNA extraction and assayed by real-time qRT-PCR.

### Statistical analysis

If not indicated in the text, analysis was performed by one-way pairwise ANOVA test. The *P*-values of <0.05 are considered as statistically significant.

## Figures and Tables

**Table 1a:** VPA treatment responsive selected GO categories for downregulated differentially expressed genes (*P*<0.05)

**Term**	**Count**	***P*-value**	**Genes**
Regulation of transcription, DNA-dependent	105	4.82E–06	*REST, RORA, ZNF254, HOXC6, TDGF3, GATA6, TDGF1, OLIG2, MLL3, PITX2, NODAL, EMX2, ZNF814, EOMES, SUZ12, HES5, SOX7, LIN28A, NR2C2, ARX, FOXH1, LHX9, DNMT3A, SALL4, FOXA2, NR6A1, PAX3, GLI2, POU5F1, HEY2, ZNF720, SOX10, NANOG, DMRT3, OTX2, RUNX1T1, SPEN, HAND1, ISL1, POU5F1B, ATRX, RBAK, THRAP3, PBX2*
Embryonic morphogenesis	26	3.44E–04	*RBP4, FOXA2, PAX3, GLI2, TDGF3, SPRY2, HAND1, PRKRA, TDGF1, CEP290, NODAL, EOMES, PCDH8, CFC1, DKK1, SALL4, GNAQ, HOXB6, LRP6, PTCH1, MAB21L2, PBX2*
Lung development	11	0.004697	*SPRY2, RBP4, FOXA2, PDGFA, GATA6, DHCR7, NODAL, VEGFA, GLI2, FOXP1, KDR*
Regulation of neurogenesis	13	0.026608	*PHOX2B, NBN, BMP2, FOXA2, REST, ISL1, GLI2, RUFY3, HES5, CCND2, NUMB, NEFM, ASPM*
Forebrain development	16	7.60E–04	*OTX2, EMX2, GLI2, ISL1, AHSG, ARX, SALL3, ATRX, FEZF2, DKK1, GNAQ, NUMB, PAFAH1B1, LRP8, RELN, ASPM*
Neuron differentiation	32	8.12E–04	*FGFR1, FOXA2, ONECUT2, GRIN3A, PAX3, RORA, GLI2, PTEN, CXCL12, KLHL1, ARX, KAL1, NUMB, CEP290, OLIG1, PAFAH1B1, OLIG2, RET, BHLHE22, OTX2, EMX2, LIFR, ISL1, CDKN1C, SALL3, FEZF2, EPHA4, ADM, GNAQ, HES5, VEGFA, RELN*
Cerebral cortex development	6	0.008017	*ARX, EMX2, LRP8, PAFAH1B1, RELN, AHSG*
Telencephalon development	7	0.038287	*ARX, SALL3, EMX2, LRP8, PAFAH1B1, RELN, AHSG*

The selected genes were represented corresponding to the respective GO

**Table 1b:** VPA treatment responsive selected GO categories for upregulated differentially expressed genes (*P*<0.05)

**Term**	**Count**	***P*-value**	**Genes**
Neuron development	24	0.002116771	*EGFR, ITGA1, NTNG2, NRXN1, PRKG1, SLIT1, GPR98, ALCAM, EFHD1, NCAM2, EPHA7, SLITRK4, SLITRK3, S100B, CRB1, UNC5A, SEMA3A, BMP7, SNAP25, C1ORF187*
Axonogenesis	16	0.003498804	*UCHL1, NTNG2, NRXN1, SLIT1, ALCAM, NCAM2, ATOH1, EPHA7, SLITRK4, SLITRK3, S100B, UNC5A, SEMA3A, BMP7, SNAP25, C1ORF187*
Vasculature development	17	0.016483196	*CAV1, HTATIP2, FOXO1, ARHGAP24, CITED1, ANXA2, ANXA2P2, PROK2, HOXA3, ID1, CTGF, PLXDC1, CASP8, SEMA3C, EGF, THBS1, SCG2*
Skeletal system development	21	0.009263376	*EGFR, COL2A1, SPARC, ANXA2, HOXB4, DLX2, COL9A2, SOST, CHRDL1, HOXA3, HOXC4, NKX3-2, COL12A1, GPNMB, BMP7, COL11A1, BMP8B, BMP5, BMP6*

The selected genes were represented corresponding to the respective GO

## Abbreviations

MOs: antisense morpholino oligonucleotides
bFGF: basic fibroblast growth factor
BMC: benchmark concentration
BPs: biological processes
DAVID: database for annotation, visualization and integrated discovery
DEGs: differentially expressed genes
DMSO: dimethyl sulfoxide
DNMTs: DNA methyl transferases
EBs: embryoid bodies
ESCs: embryonic stem cells
GO: gene ontology
HDACi: histone deacetylase inhibitors
hESCs: human embryonic stem cells
iPSC: induced pluripotent cells
IC: inhibitory concentration
MBD: methyl-CpG binding domain
miRNAs: microRNAs
PCA: principal component analysis
SAHA: suberoylanilide hydroxamic acid
TSA: trichostatin A
VPA: valproic acid
VIM: vimentin
