# Supplementary figures and images for: Neuronal developmental gene and miRNA signatures induced by histone deacetylase inhibitors in human embryonic stem cells
Source: Cell Death Dis. 2015 May 7;6(5):e1756–. doi: 10.1038/cddis.2015.121 (PMC4669700; doi:10.1038/cddis.2015.121)

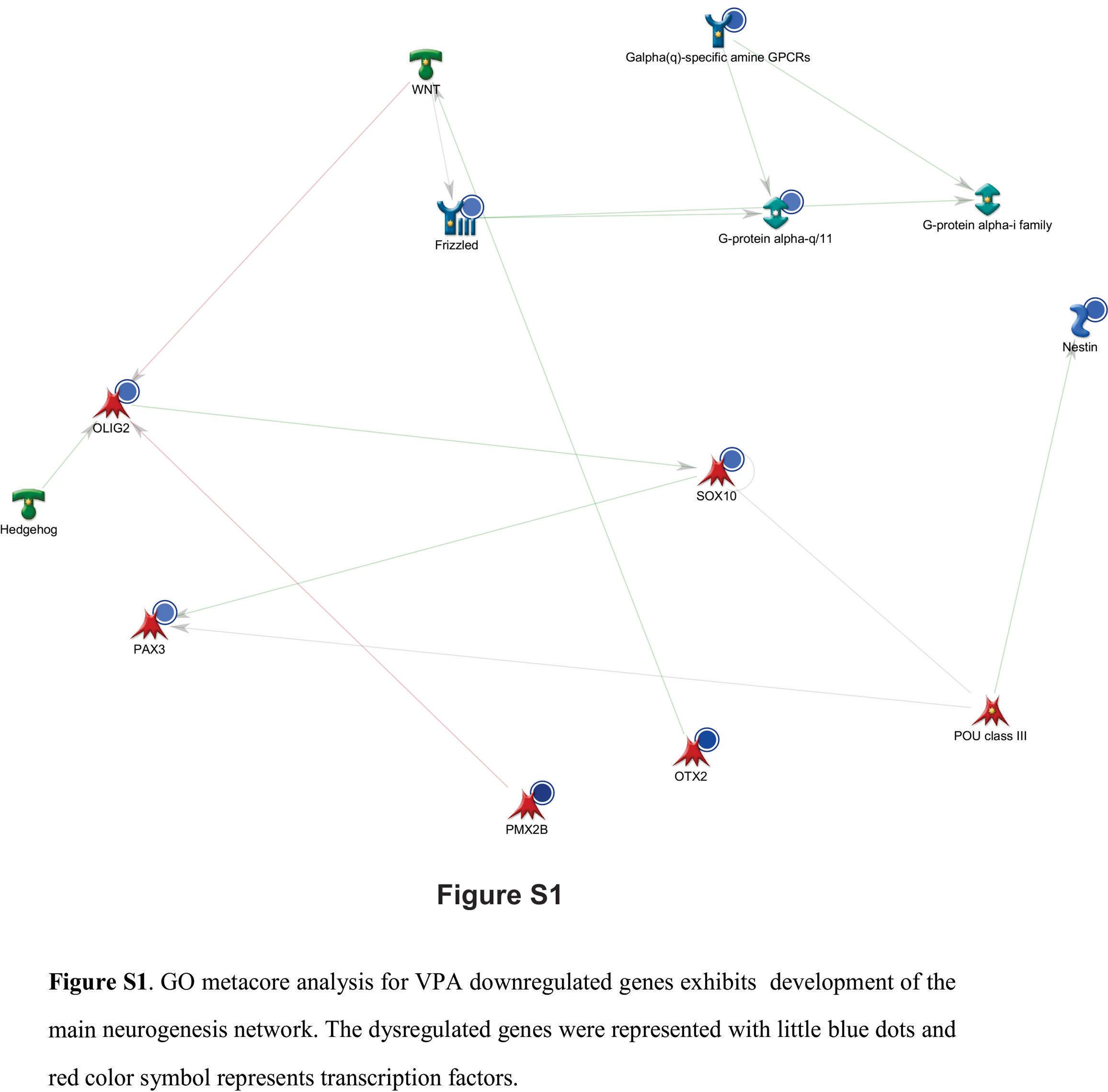

Supplement: Supplementary Figure 1 [file cddis2015121x1.tif]
