# Supplementary material for: Neuronal developmental gene and miRNA signatures induced by histone deacetylase inhibitors in human embryonic stem cells
Source: Cell Death Dis. 2015 May 7;6(5):e1756–. doi: 10.1038/cddis.2015.121 (PMC4669700; doi:10.1038/cddis.2015.121)
Supplement: Supplementary Table Legends [file cddis2015121x3.doc]

**Table S1**. Primers used for RT-qPCR.

**Table 2S (A-C)**. The differentially regulated genes were derived using one-way ANOVA for each compound treatment compared to their respective control. **A**, VPA, **B**, SAHA, **C**, TSA.

**Table S3**. The differentially regulated miRNA genes were derived using one-way ANOVA for VPA treatment compared to control.

**Table S4 (A-E)**. DAVID GO analysis for differentially regulated genes for each compound up and down regulated genes separately. Lower number of regulated genes did not provided any GOs. **A**, VPA-downregulated, **B**, VPA-upregulated, **C**, SAHA-downregulated, **D**, SAHA-upregulated, **E**, TSA-upregulated.
